# Tea Polyphenols in Promotion of Human Health

**DOI:** 10.3390/nu11010039

**Published:** 2018-12-25

**Authors:** Naghma Khan, Hasan Mukhtar

**Affiliations:** 4385 Medical Sciences Center, 1300 University Ave, Dept. of Dermatology, University of Wisconsin-Madison, Madison, WI 53706, USA; hmukhtar@dermatology.wisc.edu

**Keywords:** cancer, EGCG, diseases, green tea, tea polyphenols

## Abstract

Tea is the most widely used beverage worldwide. Japanese and Chinese people have been drinking tea for centuries and in Asia, it is the most consumed beverage besides water. It is a rich source of pharmacologically active molecules which have been implicated to provide diverse health benefits. The three major forms of tea are green, black and oolong tea based on the degree of fermentation. The composition of tea differs with the species, season, leaves, climate, and horticultural practices. Polyphenols are the major active compounds present in teas. The catechins are the major polyphenolic compounds in green tea, which include epigallocatechin-3-gallate (EGCG), epigallocatechin, epicatechin-3-gallate and epicatechin, gallocatechins and gallocatechin gallate. EGCG is the predominant and most studied catechin in green tea. There are numerous evidences from cell culture and animal studies that tea polyphenols have beneficial effects against several pathological diseases including cancer, diabetes and cardiovascular diseases. The polyphenolic compounds present in black tea include theaflavins and thearubigins. In this review article, we will summarize recent studies documenting the role of tea polyphenols in the prevention of cancer, diabetes, cardiovascular and neurological diseases.

## 1. Introduction

The beverage tea is made from the infusion of the leaves of *Camellia sinensis*. The world’s tea consumption is highest for black tea, followed by green tea, oolong tea, and white tea. Black tea is made by crushing and drying fresh tea leaves to effect fermentation prior to final processing and is consumed usually in the United States, Europe, Africa, and India. During fermentation, some of the catechins combine to form complex theaflavins and other flavonoids, which offer characteristic taste and color to black tea. To prevent fermentation, green tea is prepared when the fresh leaves are processed swiftly and the oolong tea is partially fermented. 

Tea possesses antioxidant properties with traces of proteins, carbohydrates, amino acids, lipids, vitamins and minerals. It also contains an extensive range of chemical compounds, but mainly polyphenols account for the aroma and beneficial health effects of tea. The polyphenols in green tea are credited with its beneficial properties against several diseases in many reported studies [1]. These polyphenols are present in much higher concentrations in green tea than black or oolong tea and this accounts for their antioxidant properties. The distinctive polyphenolic compounds present in green tea are called as catechins, like (-)-epigallocatechin-3-gallate (EGCG), (-)-epigallocatechin (EGC), (-)-epicatechin-3-gallate (ECG) and (-)-epicatechin (EC). EGCG account for 50–70% of catechins. EGCG is the major catechin in tea and accounts for most of the research carried out with green tea. One cup of green tea contains up to 200 mg of EGCG, which has been shown to have chemopreventive/chemotherapeutic effects against several types of cancers [2,3]. Proper drinking of green tea is three to five cups per day, which accounts for a minimum of 250 mg of catechins per day [4]. Several in-vitro and in-vivo studies have reported the antioxidant effects of GTP. We have earlier discussed the anticarcinogenic effects of green tea, its effects on various receptor tyrosine kinases, signal transduction pathways and metastasis [1,5,6]. In this article, we present recent scientific evidences, for the anticarcinogenic effect of green tea and its role in diabetes, cardiovascular and neurological diseases. Anticarcinogenic effects of tea polyphenols and their mechanisms in different cancer types are shown in Table 1.

## 2. Green Tea Polyphenols and Lung Cancer

### 2.1. In-Vitro Studies

Lung cancer is the primary cause of cancer-related deaths worldwide, and non-small cell lung cancer (NSCLC) accounts for 80% of lung cancer cases. Recently, it was reported that EGCG inhibited programmed cell death ligand 1 (PD-L1) expression in NSCLC cells, induced by both interferon (IFN)-γ and epidermal growth factor (EGF) [7]. In NSCLC cells, pretreatment with EGCG and green tea extract (GTE) caused decrease in the mRNA and protein levels of IFN-γ-induced PD-L1, through inhibition of Janus kinase (JAK)/signal transducers and activators of transcription (STAT) signaling. Pre-treatment with EGCG also caused decrease in EGF-induced PD-L1 expression through inhibition of EGF receptor (EGFR)/Akt signaling [7]. 

ECG, a natural polyphenolic component of green tea, inhibited the invasion of NSCLC cells by suppressing the levels of matrix metalloproteinase (MMP)-2 and urokinase type plasminogen activator (uPA) [8]. It also reversed the transforming growth factor (TGF)-β1-induced epithelial-mesenchymal transition (EMT) and upregulated E-cadherin, while it caused the inhibition of mesenchymal markers, such as fibronectin and p-FAK. Subcutaneous inoculation of ECG also inhibited the tumor growth of NSCLC cells in xenograft model [8]. The clinical efficacy of tea polyphenols depends on efficient delivery and bioavailability [9,10,11]. Atomistic Molecular Dynamics simulations have shown that EGCG naturally binds to the hydrophilic regions of phospholipids, positioning mostly at the interface between water and lipid phases [12]. EGCG was encapsulated inside anionic liposomes made of 1-palmitoyl-2-oleoyl-sn-glycero-3-phosphocholine, 1,2-dioleoyl-sn-glycero-3-phosphoethanolamine and cholesteryl hemisuccinate to escalate its delivery. The ability of these liposomes to contrast H_2_O_2_-induced cell death was investigated in human retinal cells. Mitochondria were better preserved in cells treated with liposomes as compared to those treated with free EGCG. It was concluded that the produced formulation improved the efficacy of EGCG and could be used for diseases caused by oxidative damage [13].

Using nanochemoprevention, tea polyphenols, like theaflavin (TF) and EGCG were encapsulated in a biodegradable nanoparticulate formulation based on poly(lactide-co-glycolide) (PLGA) [14]. The ability of both bulk TF/EGCG and TF/EGCG-loaded PLGA-NPs to inhibit proliferation of lung carcinoma, cervical carcinoma and acute monocytic leukemia cells was determined. There was about three to seven fold reduction in IC_50_ doses on treatment with TF/EGCG-loaded NPs when compared with bulk doses of polyphenols. There was lack of toxicity of PLGA-NPs as evidenced by the treatment of cells. TF/EGCG-NPs were also more effective than bulk TF/EGCG in sensitizing lung cancer cells to cisplatin-induced apoptosis. The combination of TF/EGCG-NPs and cisplatin caused inhibition of NF-κB activation, cyclin D1, MMP-9 and vascular endothelial growth factor (VEGF). A proteomic-based approach was employed to identify proteins modulated by EGCG in A549 lung cancer cells [15]. Hepatoma-derived growth factor (HDGF) is considered as a therapeutic target in lung cancer. Treatment with EGCG caused three-fold suppression of HDGF and downregulation of HDGF by EGCG was confirmed using anti-HDGF antibodies in lung cancer cell lines. EGCG treatment also induced synergistic effect with cisplatin in causing lung cancer cell death and increased cytotoxicity was also noted in HDGF-silenced cells. Induction of apoptosis, disruption of the mitochondrial membrane potential, and activation of caspase-3 and -9 were linked to cell death. It was concluded that decreasing the levels of HDGF by treatment with EGCG may signify a novel approach in treatment of lung cancer. In addition, EGCG induced a marked synergistic effect with cisplatin in cell death. Consistently, an enhanced cytotoxicity in HDGF-silenced cells was also found. Cell death was associated to increased apoptosis, disruption of the mitochondrial membrane potential, and activation of caspase-3 and -9 [15]. Treatment of human lung cancer cells with EGCG caused inhibition of anchorage-independent growth and induction of G0/G1 phase cell-cycle arrest [16]. Suppression of EGFR pathway was found to be involved in the anticancer efficacy of EGCG. Short term exposure of human lung cancer cells with EGCG decreased EGF-induced EGFR, AKT and activation of ERK1/2. Chronic treatment with EGCG caused inhibition of total and membranous EGFR expression and decreased nuclear localization of EGFR with downregulation of cyclin D1. Also, sensitivity of lung cancer cells to EGCG was decreased on knockdown of EGFR, confirming that EGFR signaling may be involved in the anticancer activity of EGCG in human lung cancer cells [16]. The involvement of AP-1 in GTP-induced tumor inhibition was investigated in human NSCLC cell line H1299 and mouse SPON 10 cells [17]. These cell lines displayed high constitutive AP-1 activity and cell growth was inhibited when TAM67 expression was induced with doxycycline and connected with inhibited AP-1 activity. RNA-seq was used to define the global transcriptional effects of AP-1 inhibition and to elucidate the possible involvement of AP-1 in GTP-induced chemoprevention. AP-1 was identified as a key transcription regulator. In TAM67 expressing H1299 cells, 293 genes were downregulated on treatment with polyphenon E (PPE), and 10% of them had a direct AP-1 binding site, suggesting that AP-1 is the target of PPE. Regarding the inhibition of AP-1, chemopreventive properties of PPE were lost, signifying that AP-1 pathway is targeted by GTP [17].

### 2.2. In-Vivo Studies

In 4-(methylnitrosamino)-1-(3-pyridyl)-1-butanone (NNK)-induced lung cancer model, 0.3% GTE in drinking water decreased the tumor multiplicity and the percentage of PD-L1 positive cells. Thus, it was shown that EGCG acts as an alternative immune checkpoint inhibitor [7]. The PLGA-NPs in combination with cisplatin decreased tumor volume and increased longevity in mice bearing Ehrlich’s ascites carcinoma cells. Thus, it was shown that EGCG and TF-NPs were more effective than bulk EGCG/TF [14]. Functional genomic approaches were used to explain the role of microRNA in the inhibition of tobacco carcinogen-induced lung tumors in A/J mice by EGCG [18]. Modest changes were noted in the expression levels of 21 microRNAs and by comparing these microRNAs with the mRNA expression profiles using the computation methods, 26 potential targeted genes of these microRNAs were identified. It was noted that Akt, NF-κB, MAP kinases and cell cycle pathways were modulated after treatment with EGCG, demonstrating that the miRNA-mediated regulation was involved in the anti-cancer activity of EGCG in-vivo [18].

### 2.3. Studies in Humans

A cross-sectional survey with the use of data from the Korean National Health and Nutritional Examination Survey collected between 2008 and 2015 reported an association between green tea intake and chronic obstructive lung disease (COPD) [19]. To examine the association between the frequency of green tea intake and risk of COPD, multiple linear and logistic regression models were used after adjusting for age, sex, body mass index, smoking status, alcohol consumption, physical activity, and socioeconomic status. It was reported that there was decrease in the incidence of COPD with an increase in the consumption of green tea from never to ≥2 times per day, highlighting that the intake of green tea is associated with a reduced risk of COPD in Korean populations [19]. 

## 3. Green Tea Polyphenols and Colorectal Cancer

### 3.1. In-Vitro Studies

Colorectal cancer (CRC) is regarded as one of the most prevalent form of cancer because of its predominant incidences in both males and females worldwide. Recently, it has been reported that treatment of CRC cells with EGCG and radiation augmented the sensitivity to radiation by inhibition of cell proliferation and induction of nuclear factor (erythroid-derived 2)-like 2 (Nrf2) nuclear translocation and autophagy. Treatment with combination of EGCG and radiation also induced the expression of LC3 and caspase-9 mRNA [20]. Increased expression of the enhancer of zeste homologue 2 (EZH2) is associated with disease progression and a poorer prognosis in several types of cancers. Ying et al., investigated whether EGCG could be a potential EZH2 inhibitor, with a mechanism similar to that of GSK343 (EZH2 inhibitor) in CRC cells [21]. Levels of the EZH2 were found to be significantly higher in CRC tissues compared to normal adjacent tissues and different human CRC cell lines displayed contradictory expression of the EZH2 protein levels. In RKO CRC cells, EGCG and GSK343 inhibited proliferation, invasion and migration and caused suppression of the protein expression of trimethylated lysine 27 on histone H3 (H3K27me3), which may be caused by the loss of the enzymatic function of EZH2. There was synergistic effect of EGCG and GSK343 on the growth of CRC cells at low doses. Both EGCG and GSK343 caused G0/G1 phase arrest in cell cycle, signifying that EGCG and GSK343 work through a common mechanism of action in CRC cells [21]. Cancer stem cells (CSCs) are an infrequent subpopulation of cancer cells that demonstrates the abilities of self-renewal and multipotent differentiation and have an important part in initiation and development of tumors [22,23]. Treatment with EGCG inhibited the spheroid formation competency of CRC cells, expression of colorectal CSC markers, inhibition of cell proliferation, induction of apoptosis accompanied by downregulation of the activation of Wnt/β-catenin pathway [24]. The platinum-based chemotherapy treatments are used extensively for the treatment of CRC, but they have various adverse cytotoxic effects. A combination of EGCG with cisplatin or oxaliplatin was used to minimize the side effects of platinum-based therapy [25]. Treatment of human CRC cells with EGCG plus cisplatin or oxaliplatin showed a synergistic effect on the inhibition of cell proliferation and induction of cell death. EGCG treatment also improved the effect of cisplatin and oxaliplatin-induced autophagy as shown by the accumulation of LC3-II protein, rise of acidic vesicular organelles and the formation of autophagosome. These findings recommend that combination of EGCG with cisplatin or oxaliplatin could decrease cytotoxicity in CRC cells through autophagy related pathways [25]. The chemosensitizing effects of EGCG in 5-fluorouracil (FU)-resistant (5-FUR) CRC cells and spheroid-derived CSCs (SDCSCs) were investigated in a recent study [26]. Treatment with EGCG boosted 5-FU induced cytotoxicity and suppressed proliferation in 5-FUR cell lines through enhancement of apoptosis and cell cycle arrest. The higher spheroid forming capacity was shown in 5-FUR cells as compared to parental cells, representing higher CSC population. Treatment with EGCG led to suppression of SDCSC formation and enhanced 5-FU sensitivity to SDCSCs. EGCG also inhibited pathways targeted in 5-FUR CRC cells such as Notch1, Bmi1, Suz12, and Ezh2, and upregulated self-renewal suppressive-miRNAs, miR-34a, miR-145, and miR-200c [26]. 

### 3.2. In-Vivo Studies

The inhibitory effects of orally administered PPE on colon carcinogenesis in azoxymethane-treated rats have been reported. PPE is a defined GTP preparation containing about 65% EGCG and less than 0.1% caffeine. Treatment with PPE in diet significantly increased the plasma and colonic levels of tea polyphenols, reduced tumor multiplicity, tumor size and decreased the incidence and multiplicity of adenocarcinoma. It also caused decrease in the levels of proinflammatory eicosanoids, prostaglandin E2 and leukotriene B4. PPE treatment also lowered β-catenin nuclear expression and caused induction of apoptosis and augmented expression levels of RXR α, β and γ in adenocarcinomas [27]. Treatment with EGCG caused inhibition of tumor growth in a SDCSC xenograft model. It was concluded from the study that EGCG may assist as an adjunctive treatment to conventional chemotherapeutic drugs in CRC patients [26].

### 3.3. Studies in Humans

In a randomized clinical trial, the effect of green tea extract (GTE) supplements on metachronous colorectal adenoma and cancer in the Korean population was determined [28]. Patients who had undergone complete removal of colorectal adenomas by endoscopic polypectomy were divided into two groups. One group was control and the other was given 0.9 g GTE/day for 12 months. It was found that the incidence of metachronous adenomas at the end-point colonoscopy was higher in control group (42.3%) than GTE-supplemented group (23.6%). Relapsed adenoma was also decreased in the GTE group as compared to the control group, although no differences were noted between two groups in regards to body mass index, dietary intakes, serum lipid profiles, fasting serum glucose and serum C-reactive protein levels. It was concluded that GTE supplements were promising for the chemoprevention of metachronous colorectal adenomas in Korean patients [28]. 

## 4. Green Tea Polyphenols and Skin Cancer

### 4.1. In-Vitro Studies

Skin cancer can be classified as basal cell carcinoma, squamous cell carcinoma, and melanoma, according to histological characteristics [29]. The effect of tea polyphenols on Toll-like receptor 4 (TLR4) in melanoma cell lines has been reported recently [30]. Treatment of melanoma cell lines (B16F10 and A375) with tea polyphenols inhibited the proliferation, migration and invasion ability of melanoma cells dose and time dependently. As compared with normal skin cells, TLR4 was greatly expressed in melanoma cells. Treatment with tea polyphenols inhibited TLR4 expression both in normal melanomas and in stimulated melanomas by TLR4 agonist lipopolysaccharides. The inhibition of TLR4 in melanoma cell lines inhibited cell proliferation, migration, and invasion, and blocking the expression of 67LR eliminated the effects of tea polyphenols on TLR4 [30]. It has been shown that the physiological doses of EGCG (0.1–1 μM) inhibited the proliferation of human metastatic melanoma cell lines [31]. Treatment with EGCG also inhibited NF-κB activity and IL-1β secretion, which was related with downregulation of NLRP1 and a decrease in the activation of caspase-1. The inhibitory effect of EGCG on tumor proliferation was eliminated by silencing NLRP1, signifying a key role of inflammasomes in the tumor-inhibitory effect of EGCG in human melanoma cells [31]. TRAF6, a member of the tumor necrosis factor receptor-associated factor (TRAF) family, has been identified as a novel target of EGCG [32]. They employed a structure-based virtual screening to identify TRAF6 as a potential target of EGCG, and a pull-down assay revealed that EGCG directly binds to TRAF6. EGCG was found to bind with TRAF6 at the residues of Gln54, Gly55, ILe72, Cys73, Asp57 and Lys96. EGCG also inhibited the E3 ubiquitin ligase activity of TRAF6 both in-vitro and in-vivo. EGCG treatment blocked the regulation of NF-κB pathway activation by TRAF6 and inhibits melanoma cell growth, invasion and migration. Therefore, this study suggests that EGCG is an E3 ubiquitin ligase inhibitor and inhibits melanoma cell growth and metastasis by targeting TRAF6 [32]. The 67-kDa laminin receptor (67LR) has been identified as a cell surface receptor of EGCG and has a role in its anticancer effects. EGCG inhibited melanoma tumor growth by activating 67-kDa laminin receptor (67LR) signaling [33]. Treatment of melanoma cells with EGCG up-regulated miRNA-let-7b expression through 67LR, which in turn, caused downregulation of high mobility group A2 (HMGA2), a target gene connected to tumor progression. It was demonstrated that the upregulation of let-7b expression by EGCG followed activation of 67LR-dependent cAMP/protein kinase A (PKA)/protein phosphatase 2A (PP2A) signaling pathway [33].

### 4.2. In-Vivo Studies

Oral gavage treatment with tea polyphenols inhibited B16F10 melanoma cells growth in-vivo. Treatment with tea polyphenols decreased the tumor size and tumor volume along with the inhibition of TLR4 protein expression as compared with the control group [30]. The effects of tea polyphenols against UVB-induced skin cancer has been reported [34]. GTP can be easily oxidized in the environment and slowly lose their activity. Preserving the activity of GTP for topical formulations is challenging as browning takes place during the storage of skin cream supplemented with green tea catechins. Therefore, Li et al., demonstrated the stabilizing effect of carboxymethyl cellulose sodium (CMC-Na) on GTP under aqueous conditions [35]. Topical application of GTP, emulsified in CMC-Na had a strong photoprotective effect against acute UVB induced photodamage in hairless mice skin. It was reported that 93% of GTP was preserved after 8 h of incubation at 50 °C with CMC-Na, whereas in the absence of CMC-Na, only 61% was preserved. There was also inhibition of acute UVB-induced infiltration of inflammatory cells, increase of skin thickness, depletion of antioxidant enzymes and lipid oxidation, and induction of nuclear accumulation of Nrf2 in mice skin on topical treatment of emulsified GTP [35]. 

### 4.3. Studies in Humans

In a case-control study, data from 767 non-Hispanic Whites under age 40 was evaluated to understand the effects of tea, coffee, and caffeine on the early-onset of basal cell carcinoma (BCC). Inverse relationship was found to be associated with combined regular consumption of caffeinated coffee plus hot tea with early-onset of BCC. There was 43% reduced risk of BCC in people consuming the highest category of caffeine from these sources as compared with non-consumers. This study concluded that there was a modest protective effect for caffeinated coffee plus tea in relation to early-onset BCC [36]. 

## 5. Green Tea Polyphenols and Prostate Cancer

### 5.1. In-Vitro Studies

Prostate cancer (PCa) is the most commonly diagnosed malignancy in males and we have earlier reported in detail the effects of GTP on various signaling pathways in PCa [37,38], and its preclinical and clinical effects [1,39]. Polymeric EGCG-encapsulated nanoparticles (NPs) targeted with small molecular entities that were able to bind to prostate specific membrane antigen (PSMA) were developed [40]. Increased anti-proliferative activity and induction of apoptosis in PCa cell lines was observed on treatment with EGCG encapsulating NPs compared to the free EGCG [40]. We have earlier reported the synthesis, characterization and efficacy assessment of a nanotechnology-based oral formulation of chitosan nanoparticles with a size in the range of 150–200 nm diameter encapsulating EGCG (Chit-nanoEGCG) for the treatment of PCa in a preclinical setting [41]. We synthesized nanoparticles made up of the natural biopolymer chitosan with encapsulated EGCG, which appeared to be stable in the acidic environment of the stomach and prevented release of EGCG in the stomach. These nanoparticles showed a slow release of EGCG in acidic pH (simulated gastric juice) and faster release in simulated intestinal fluid (neutral pH) [41]. It has been shown that there was decrease in PCa cell survival and induction of apoptosis with a low dose of 1 μM EGCG [42]. Treatment with EGCG also boosted the capacity of cisplatin to promote apoptosis, and EGCG, both alone and in combination with cisplatin, stimulated the expression of the pro-apoptotic splice isoform of caspase-9 in PCa cells [42]. In human PCa cells, GTP and EGCG activated p53 through acetylation at the Lys373 and Lys382 residues by inhibiting class I Histone deacetylases (HDACs) [43]. There was dose- and time-dependent inhibition of class I HDACs (HDAC1, 2, 3 and 8) on treatment of PCa cells with GTP (2.5–10 μg/mL) and EGCG (5–20 μM), while loss of p53 acetylation at both the sites was observed on withdrawal of treatment with GTP/EGCG. Increased expression of p21/WAF1 was also noted on treatment with GTP/EGCG in PCa cells. The increased GTP/EGCG-mediated p53 acetylation improved its binding on the promoters of p21/WAF1 and Bax. This in turn was connected with an increase in the accumulation of cells in the G0/G1 phase of the cell cycle and induction of apoptosis [43]. The effect of epicatechin (EC), epigallocatechin (EGC) and EGCG (EGCG) on the regulation of androgen receptor acetylation in androgen-dependent PCa cells was demonstrated by histone acetyl-transferase (HAT) activity [44]. Treatment with EC, EGC and EGCG caused PCa cell death, inhibited agonist-dependent androgen receptor (AR) activation and AR-regulated gene transcription. EGCG was the most potent HAT inhibitor among all other catechins and it downregulated AR acetylation. In the presence of the agonist, there was inhibition of AR protein translocation to the nucleus from cytoplasmic compartment [44].

### 5.2. In-Vivo Studies

In mouse xenograft model of prostatic tumor, nanoformulated EGCG displayed better efficacy than native EGCG and there was 30% tumor growth inhibition in EGCG-treated groups whereas 55 and 60% tumor growth inhibition on treatment with non-targeted- and targeted-NPs, respectively, at the end of the study [40]. It has been reported that certain stages are more or less sensitive to EGCG and that sensitivity is related to heat shock protein 90 (HSP90) inhibition in non-tumorigenic (BPH-1), tumorigenic (BCaPT1, BCaPT10) and metastatic (BCaPM-T10) cancer cells from a human PCa progression model [45]. Further strong cytotoxic effects were observed on the treatment of tumorigenic and metastatic cells with EGCG, novobiocin, or N-terminal inhibitor, 17-AAG. Animals given 0.06% EGCG in drinking water developed significantly smaller tumors than untreated mice when tumorigenic or metastatic cells were grown in-vivo. EGCG-Sepharose was found to bind more HSP90 from metastatic cells compared with non-tumorigenic cells and binding occurred through the HSP90 C-terminus, as determined by binding assays with EGCG-Sepharose, a C-terminal HSP90 antibody, and HSP90 mutants. EGCG, novobiocin, and 17-AAG also led to induction of changes in HSP90-client proteins in non-tumorigenic cells and larger differences in metastatic cells, suggesting that EGCG preferentially targets cancer cells and prevents a molecular chaperone supportive of the malignant phenotype [45].

Chit-nanoEGCG treatment of athymic nude mice subcutaneously implanted with PCa cells caused significant inhibition of tumor growth and secreted prostate-specific antigen (PSA) levels compared with EGCG and control groups. There was also induction of poly (ADP-ribose) polymerases (PARP) cleavage, increase in the protein expression of Bax with decrease in Bcl-2, activation of caspases and decrease in Ki-67, proliferating cell nuclear antigen (PCNA), CD-31 and vascular endothelial growth factor (VEGF) in tumor tissues of mice treated with Chit-nanoEGCG, as compared with groups treated with EGCG and control group [41]. This study addressed concerns related to bioavailability of EGCG and suggested that this nanoformulation also has the potential to be used as a carrier system for many of the bioactive compounds that have sensitivity to acidic pH [41]. 

### 5.3. Studies in Humans

The relationship between prostate cancer (PCa) risk and habitual green tea intake was investigated among Chinese men in Hong Kong [46]. The 404 PCa patients and 395 controls were recruited in the study from the same hospital that had complete data on habitual tea consumption of green, oolong, black and pu’er tea. Habitual green tea drinking was reported in a total of 32 cases and 50 controls, while a modest excess risk was detected among the habitual pu’er tea drinkers. An inverse gradient of PCa risk with the increasing consumption of EGCG was observed due to lower intake of EGCG among PCa patients than the controls. It was concluded that there is an inverse association of PCa risk among Chinese men in Hong Kong with green tea consumption and EGCG intake [46]. In a double-blind, placebo-controlled study, sixty volunteers with high-grade prostate intraepithelial neoplasia (HGPIN), without any given therapy was enrolled to determine whether the administration of green tea catechins (GTCs) could stop malignancy in men at high-risk [47]. Volunteers were given daily treatment of three GTCs capsules, 200 mg each. It was noted that only one tumor was diagnosed among the 30 GTCs-treated men after 1 year, as compared with nine cancers among the 30 placebo-treated men. There was not much effect on total prostate-specific antigen between the two arms, but lower values were recorded in GTCs-treated men with respect to placebo-treated ones. There was improvement in International Prostate Symptom Score and quality of life scores of GTCs-treated men with coexistent benign prostate hyperplasia with no significant side effects. Lower urinary tract symptoms also decreased on administration of GTCs, signifying that green tea could also be beneficial for benign prostate hyperplasia [47]. The role of GTCs for prostate cancer chemoprevention was further investigated in a randomized, double-blind, placebo controlled trial. PPE, as a standardized formulation of GTCs containing 400 mg EGCG/day was given to men with HGPIN and/or atypical small acinar proliferation (ASAP) [48]. The primary endpoint of the study was a comparison of the cumulative one-year PCa rates on the two study arms and there were no differences in the number of PCa cases. In a pre-specified secondary analysis performed in men with HGPIN without ASAP at baseline, a decrease in the composite endpoint of PCa plus ASAP was observed for the PPE arm. In addition, fewer men with HGPIN without ASAP at baseline were subsequently diagnosed with ASAP on the PPE than on the placebo arm. It was concluded that daily consumption of a standardized, decaffeinated catechins mixture containing 400 mg EGCG/day for 1 year accumulated in plasma and was well tolerated but did not lessen the likelihood of PCa in men with baseline HGPIN or ASAP [48].

## 6. Green Tea Polyphenols and Breast Cancer

### 6.1. In-Vitro Studies

Breast cancer is the most commonly diagnosed cancer and the main reason of cancer-related deaths among women globally. The anticancer effects of EGCG in breast cancer were investigated both in-vitro and in-vivo, based on its effect on tumor glucose metabolism [49]. Treatment of breast cancer 4T1 cells with EGCG inhibited cell growth and induced apoptosis as shown by activation of caspases-3, -8 -9, modulation of apoptotic related genes and promotion of mitochondrial depolarization. EGCG treatment also inhibited the activity of the enzymes hexokinase, phosphofructokinase and lactic dehydrogenase, enzymes related to the glycolytic pathway, specifying that modulating glucose metabolism plays an important part in the anticancer effects of EGCG [49]. Treatment with EGCG inhibited the MDA-MB-231 cell-viability, expression of β-catenin, phosphorylated Akt and cyclin D1. This study suggested that EGCG inhibits the growth of breast cancer cells through the inactivation of the β-catenin signaling pathway [50]. The effects of EGCG on proliferation and apoptosis of T47D estrogen receptor α-positive breast cancer cells, and compared with tamoxifen were reported recently. Treatment of cells with EGCG decreased cell viability in a dose and time-dependent manner. EGCG treatment of cells significantly increased PTEN, caspases-3 and -9, decreased AKT and increased Bax/Bcl-2 ratio, almost similar to tamoxifen [51]. The stability of EGCG in solutions of different pH was investigated to define the pH range of stability of EGCG under room temperature conditions. Very low stability profile of EGCG at physiological pH was observed with rapid degradation under alkaline conditions. Hence, EGCG was encapsulated in solid lipid nanoparticles (SLN) for enhancing the stability and anticancer activity. SLN control the release of encapsulated drug and consequently, prevent the premature degradation of encapsulated drug in the biological system. EGCG and EGCG loaded nanoparticles (EGCG-SLN) were compared by cellular proliferation assay in MDA-MB-231 human breast cancer and DU-145 PCa cell lines. The cytotoxicity of EGCG-SLN was found to be 8.1 times higher against human breast cancer cells and 3.8 times higher against human PCa cells, as compared with pure EGCG [52].

### 6.2. In-Vivo Studies

The effects of a nutrient mixture containing ascorbic acid, lysine, proline and green tea extract were investigated in a model of metastatic breast cancer. Treatment with nutrient mixture inhibited tumor weight and burden of metastatic breast tumors and also decreased lung metastasis, as compared to control mice. There was also decrease in the metastasis to liver, spleen, kidney and heart with NM treatment, suggesting that nutrient mixture may be explored further for the treatment of breast cancer [53].

### 6.3. Studies in Humans

Among patients who underwent surgery at Chonbuk National University Hospital, Jeonju, Korea, for primary breast cancers, 74 breast cancer patients were identified and admitted in the study to investigate the expression profiles of the β-catenin signaling pathway in breast cancer patients. The β-catenin expression was analyzed according to the clinicopathological factors of female breast cancer patients diagnosed with invasive ductal carcinoma. It was found that β-catenin was expressed at higher levels in breast cancer tissue than in normal tissue. β-catenin expression was related with lymph node metastasis, tumor-node-metastasis stage and estrogen receptor status. [50]. In a randomized phase II controlled trial, the effects of daily consumption of GTE containing 800 mg EGCG for 12 months were evaluated on changes in mammographic density (MD) measures in healthy postmenopausal women at high risk of breast cancer due to dense breast tissue. It was observed that supplementation of GTE did not significantly change percent MD (PMD) or absolute MD in all women. In younger women, GTE supplementation significantly reduced PMD as compared with the placebo, but had no effect in older women. Administration of GTE also did not prompt MD change in other subgroups of women stratified by catechol-*O*-methyltransferase genotype or level of body mass index. This study concluded that 12 months administration of a high dose of EGCG did not have a significant effect on MD measures in all women, but reduced PMD in younger women, an age-dependent effect comparable to those of tamoxifen [54]. 

### 6.4. Green Tea Polyphenols and Diabetes

Diabetes is one of the major health problems worldwide. Type-1 diabetes is not preventable and is treated by insulin supplementation. However. Type-2 diabetes can be prevented or reversed by altering diet and management of lifestyle factors. EGCG has been reported to inhibit starch hydrolysis and acted as an inhibitor by binding to the active site of α-amylase and α-glucosidase. The anti-diabetic action of EGCG was explored in high fat diet and streptozotocin (STZ)-induced type-2 diabetes. Treatment with EGCG enhanced glucose homeostasis and repressed the process of gluconeogenesis and lipogenesis in the liver. It also activated PXR/CAR, accompanied by upgrading PXR/CAR-mediated phase II drug metabolism enzyme expression in small intestine and liver, relating SULT1A1, UGT1A1 and SULT2B1b [55]. Diabetes mellitus (DM) can cause compromised wound healing by disturbing the biological mechanisms of the process. It was shown that the late wound healing in STZ-induced DM mice could be enhanced by EGCG. In the skin wounds of DM mice, EGCG treatment inhibited macrophage accumulation, inflammation response, and Notch signaling and directly bind with mouse Notch-1. Diabetic wound healing was improved on treatment with EGCG before or after the inflammation period by targeting the Notch signaling pathway, signifying that the pre-existing diabetic wound healing was enhanced by EGCG [56]. The mechanisms by which EGCG alleviates insulin resistance (IR) were explored in human hepatoma HepG2 cells. Treatment of cells with EGCG increased glucose uptake and decreased glucose content. It also reduced the intracellular levels of tumor necrosis factor-α, reactive oxygen species, malondialdehyde, with increase in antioxidant enzymes like superoxide dismutases (SOD) and glutathione peroxidase. There was also increase in the glucose transporter 2 (GLUT2) protein and its downstream proteins peroxisome proliferator-activated receptor coactivator (PGC)-1β, when cells were treated with EGCG [57]. In 3T3-L1 pre-adipocytes, EGCG has been reported to increase the activity of browning in inguinal white adipose tissue (iWAT), inhibited adipocyte differentiation and relieved TNF-α-triggered insulin resistance through the suppression of oxidative stress and regulation of mitochondrial function [58].

## 7. Green Tea Polyphenols and Cardiovascular Diseases

Cardiovascular disease is the leading cause of deaths worldwide and includes coronary heart disease (CHD), congenital heart disease, rheumatic heart disease, cerebrovascular disease and peripheral arterial disease. The relationship between plasma tea catechin and risk of stroke and CHD was investigated in a nested case-control study in men and women aged 40–69 years without history of heart disease, stroke or cancer. Participants completed a survey and donated blood samples between 1990 and 1994, and were followed-up through 2008. No significant association between plasma tea catechin and the incidence of stroke or CHD in either men or women was observed, although high plasma levels of EGCG were associated with decreased risk of stroke in non-smoking men. It was concluded that plasma tea catechin was not connected with decreased risks of either stroke or CHD, though, for male non-smokers, a protective effect of tea catechin on stroke risk was proposed [59]. The protective effect of EGCG in a mouse model of heart failure and the underlying mechanisms were investigated recently [60]. Echocardiography was employed to measure alterations in ejection fraction, left ventricular internal diastolic diameter (LVIDd) and left ventricular internal systolic diameter (LVIDs). The experiments revealed that EGCG reversed the changes in LVIDd and LVIDs, induced by establishment of the model of heart failure. There was also inhibition of myocardial fibrosis, oxidative stress, inflammatory and cardiomyocyte apoptosis, and decrease in the expression levels of collagen I and collagen III. The effect of EGCG against heart failure was diminished on treatment with TGF‑β1 inhibitor, showing that EGCG inhibited the progression and development of heart failure in mice via inhibition of myocardial fibrosis and decrease of ventricular collagen remodeling, through inhibition of TGF‑β1/smad3 signaling pathway [60].

The effects of EGCG on cardiac function by desensitization of 1-AR and GRK2 in heart failure (HF) rats were studied. Left ventricular end diastolic pressure, mean blood pressure, heart/body weight and posterior wall thickness were significantly increased in the HF group as compared to control group. Left ventricular systolic pressure, maximum rate of left ventricular pressure rise and maximum rate of left ventricular pressure fall were also lowered, whereas, treatment with EGCG recovered cardiac function by regulation of these parameters. There was decrease in the expression of 1-AR in the left ventricle tissue of HF rats and increase in expression of GRK2. Treatment with EGCG downregulated the membrane expression of GRK2 and upregulated the expression of 1-AR, suggesting it has therapeutic effects on the heart function of HF rats [61]. The protective effect of EGCG against Doxorubicin (DOX)-induced cardiotoxicity via effects on oxidative stress, inflammatory and apoptotic markers was investigated in Male Wistar rats. Treatment with EGCG was found to protect against DOX-induced ECG changes, leakage of cardiac enzymes and histopathological changes. Treatment with EGCG decreased glutathione depletion and lipid peroxidation and promotion of antioxidant enzyme activities. ErbB2 expression was reduced on treatment with DOX and it improved on treatment with EGCG. Treatment with DOX reduced expression of ErbB2, NF-κB, p53, caspases-3, -12 and basal level of Hsp70, while EGCG pretreatment significantly reversed these effects [62].

## 8. Green Tea Polyphenols and Neurological Diseases

Neurological diseases account for principal causes of disability and have high impact on the quality of life of patients and their caregivers. The effect of EGCG was investigated against neuronal injury in rat models of middle cerebral artery occlusion (MCAO). Treatment with EGCG reduced neurological function score, protected nerve cells, repressed neuronal apoptosis, and inhibited oxidative stress injury and brain injury markers level after MCAO. There was also decrease in the apoptotic rate of neurons expression, caspase-3, Bax with increase in the expression of Bcl-2. The protective effect of EGCG was decreased after administration of LY294002, a phosphoinositide 3-kinase (PI3K) inhibitor [63]. Subarachnoid hemorrhage (SAH), an exceptional subtype of stroke, has a high mortality rate. EGCG has been reported to regulate the Ca^2+^-mitochondrial dynamic axis to protect mitochondrial function after SAH. It was shown that EGCG antagonized the overloaded Ca^2+^-induced damage of mitochondrial dynamics and mitochondrial dysfunction, finally displaying neuroprotective effects after SAH. EGCG treatment improved the neurological score by reducing cell death through the Cytochrome *c*-mediated intrinsic apoptotic pathway [64]. Parkinson’s disease (PD) is a movement disorder categorized by degeneration of dopaminergic neurons and generation of intracellular deposits known as Lewy bodies and dystrophic neurites, composed primarily of alpha-synuclein (SNCA) and phosphorylated SNCA [65]. Xu et al., investigated whether EGCG inhibit the SNCA aggregation using biochemical, and tissue biological methods. They also utilized the human brain tissue for the experiment. EGCG inhibited the SNCA aggregation in a concentration dependent manner. The SNCA amino acid sites, which possibly interacted with EGCG, were detected on peptide membranes and it was suggested that EGCG inhibited the SNCA aggregation by instable intermolecular hydrophobic interactions [66].

## 9. Conclusions and Future Prospects

Tea polyphenols, especially EGCG has been the focus of research owing to it multiple protective effects against cancer and other diseases such as diabetes, neurological and cardiovascular diseases. Large amount of epidemiological and clinical studies have indicated that supplementation of green tea has significant protective effects against chronic diseases. 

Natural products with various pharmacological effects may cause drug or food interactions when administered simultaneously with narrow therapeutic index drugs. There are still many challenges for clinical application of EGCG. It has low bioavailability when given orally and it is very perplexing to derive ways to deliver EGCG effectively to target sites. The consumers should be made aware of its potential interactions with conventional medications. The tannin content of green tea interferes with intestinal absorption of some nutrients and drugs and it has inhibitory effects on CYP450 isozymes such as CYP3A4, 1A1, and 1A2. There is very restricted data on the drug and nutrient interaction of green tea in humans [67]. 

We have earlier reported in detail that EGCG modulates several signal transduction pathways and has robust cancer chemopreventive/chemotherapeutic effects [5,37,38]. It is important to recognize molecules in the cell signaling pathways which are affected on treatment with EGCG as deregulation of the network cause several chronic diseases such as cancer. The effect of EGCG on cell signaling network is evidenced by activation of cell death and induction of apoptosis in cancer cells which leads to the development of cancer progression. Tea catechins act through multiple mechanisms and these act synergistically to elicit cancer preventive and therapeutic effects. Also, tea polyphenols in combination with other drugs for chemotherapy displayed synergistic effects. Although many clinical studies have reported the beneficial effects of tea in humans [19,46,47], we are lacking in the defined evidences about the mechanisms of cancer prevention by tea in humans. To obtain more definite information, well-designed large cohort studies and human intervention trials are necessary.

## Figures and Tables

**Table 1:** Anticarcinogenic effects of tea polyphenols, reported from 2011–2018.

Target Organ	Mechanism of Action	References
Lung cancer	Decrease in the mRNA and protein levels of IFN-γ-induced PD-L1, through inhibition of JAK/ STAT signaling. Decrease in EGF-induced PD-L1expression through inhibition of EGFR/Akt signaling. Decreased tumor multiplicity in NNK-induced mice.	[7]
	In Korean population, decrease in the incidence of COPD with an increase in the consumption of green tea intake from never to ≥2 times/day	[8]
	Suppression of the levels of MMP-2 and uPA	[9]
	Upregulation of E-cadherin, inhibition of fibronectin and p-FAK. Inhibition of tumor growth in xenograft model	[10]
	Inhibition of NF-κB activation, cyclin D1, MMP-9 and VEGF on combination of EGCG and TF-nanoparticles with cisplatin	[11]
	Suppression of EGFR pathway	[13]
Colorectal Cancer	Inhibition of cell proliferation and induction of Nrf2 nuclear translocation and autophagy, expression of LC3 and caspase-9 mRNA	[15]
	Decrease in the expression of colorectal CSC markers, inhibition of cell proliferation, induction of apoptosis and downregulation of Wnt/β-catenin pathway	[19]
	Reduced tumor multiplicity, tumor size, decrease in the incidence and multiplicity of adenocarcinoma in rats. Decrease in PGE2, leukotriene B4, β-catenin nuclear expression and increase in RXR α, β and γ	[20]
Skin Cancer	Inhibition of the proliferation, migration and invasion of melanoma cells, inhibition of TLR4 expression	[25]
	Inhibition of NF-κB activity, IL-1β secretion related with downregulation of NLRP1	[28]
	Inhibition of melanoma tumor growth by activation of 67-kDa laminin receptor (67LR) signaling	[30]
Prostate Cancer	Inverse association of PCa risk among Chinese men in Hong Kong with green tea consumption and EGCG intake	[34]
	In mouse xenograft model of prostatic tumor, nanoformulated EGCG had better efficacy than native EGCG	[35]
	In xenograft study, Chit-nanoEGCG caused inhibition of tumor growth and PSA levels, induction of PARP cleavage, increase in Bax with decrease in Bcl-2, activation of caspases and decrease in Ki-67, PCNA, CD-31 and VEGF	[37]
	Inhibition of class I HDACs (HDAC1, 2, 3 and 8), arrest of cells in G0/G01 phase of cell cycle and induction of apoptosis	[39]
	Inhibition of agonist-dependent AR activation and AR-regulated gene transcription	[40]
Breast Cancer	Inhibition of cell growth, activation of caspases-3, -8 -9, promotion of mitochondrial depolarization, inhibition of the activity of the enzymes hexokinase, phosphofructokinase and lactic dehydrogenase	[41]
	Decrease in cell-viability, β-catenin, p-AKT and cyclin D1	[43]
	Increase in PTEN, caspases-3 and -9, decreased AKT and increased Bax/Bcl-2 ratio, comparable to tamoxifen	[44]

This list provides selected examples.
